# A data mining approach for classification of orthostatic and essential tremor based on MRI‐derived brain volume and cortical thickness

**DOI:** 10.1002/acn3.50947

**Published:** 2019-11-26

**Authors:** Julián Benito‐León, Elan D. Louis, Virginia Mato‐Abad, Alvaro Sánchez‐Ferro, Juan P. Romero, Michele Matarazzo, J. Ignacio Serrano

**Affiliations:** ^1^ Department of Neurology University Hospital “12 de Octubre” Madrid Spain; ^2^ Centro de Investigación Biomédica en Red sobre Enfermedades Neurodegenerativas (CIBERNED) Madrid Spain; ^3^ Department of Medicine Complutense University Madrid Spain; ^4^ Department of Neurology Yale School of Medicine Yale University New Haven Connecticut; ^5^ Department of Chronic Disease, Epidemiology Yale School of Public Health Yale University New Haven Connecticut; ^6^ Center for Neuroepidemiology and Clinical Neurological Research Yale School of Medicine Yale University New Haven Connecticut; ^7^ ISLA Faculty of Computer Science A Coruña University A Coruña Spain; ^8^ Department of Neurology HM CINAC University Hospital HM Puerta del Sur Móstoles Madrid Spain; ^9^ Research Laboratory of Electronics Massachusetts Institute of Technology Cambridge Massachusetts; ^10^ Faculty of Experimental Sciences Francisco de Vitoria University Pozuelo de Alarcón Madrid Spain; ^11^ Brain Damage Unit Hospital Beata Maria Ana Madrid Spain; ^12^ Pacific Parkinson's Research Centre and Djavad Mowafaghian Centre for Brain Health University of British Columbia Vancouver BC Canada; ^13^ Neural and Cognitive Engineering group Center for Automation and Robotics CAR CSIC‐UPM Arganda del Rey Madrid Spain

## Abstract

**Objective:**

Orthostatic tremor (OT) is an extremely rare, misdiagnosed, and underdiagnosed disorder affecting adults in midlife. There is debate as to whether it is a different condition or a variant of essential tremor (ET), or even, if both conditions coexist. Our objective was to use data mining classification methods, using magnetic resonance imaging (MRI)‐derived brain volume and cortical thickness data, to identify morphometric measures that help to discriminate OT patients from those with ET.

**Methods:**

MRI‐derived brain volume and cortical thickness were obtained from 14 OT patients and 15 age‐, sex‐, and education‐matched ET patients. Feature selection and machine learning methods were subsequently applied.

**Results:**

Four MRI features alone distinguished the two, OT from ET, with 100% diagnostic accuracy. More specifically, left thalamus proper volume (normalized by the total intracranial volume), right superior parietal volume, right superior parietal thickness, and right inferior parietal roughness (i.e., the standard deviation of cortical thickness) were shown to play a key role in OT and ET characterization. Finally, the left caudal anterior cingulate thickness and the left caudal middle frontal roughness allowed us to separate with 100% diagnostic accuracy subgroups of OT patients (primary and those with mild parkinsonian signs).

**Conclusions:**

A data mining approach applied to MRI‐derived brain volume and cortical thickness data may differentiate between these two types of tremor with an accuracy of 100%. Our results suggest that OT and ET are distinct conditions.

## Introduction

Orthostatic tremor (OT), also known as shaky‐leg syndrome,^1^ is an extremely rare, often misdiagnosed and underdiagnosed disorder affecting adults in midlife. The current view is that it may be a family of diseases, unified by the presence of leg tremor, but further characterized by etiological and clinical heterogeneity.^2^ Aside from motor manifestations, OT is associated with a number of non‐motor manifestations, including depression, cognitive dysfunction, and personality changes,^3^, ^4^ heterogeneity of pharmacological response profiles and clinical progression, and with Parkinson’s disease and other types of parkinsonism.^2^

There is some debate as to whether OT is a different condition or a variant of essential tremor (ET).^5^, ^6^ Although tremor of the legs may occur in ET, it always occurs with upper limb tremor, and at frequencies less than 12 Hz, unlike OT.^7^, ^8^ However, the main reason to consider the link between both diseases is that most patients with OT have upper limb tremor, with the proportions ranging from 77.4% to 92.3%,^9^, ^10^, ^11^ although only a few of them have a family history of ET.^11^ The question is whether those lower‐frequency arm oscillations in OT may represent a subharmonic of the higher‐frequency tremors typical of OT, spreading throughout the body or not.^12^ In favor that ET may coexist with OT, rather than being a subharmonic of the original high‐frequency tremor, “postural upper extremity tremor while seated” was documented in 22.8% of 184 patients and 12% had a family history of ET.^13^ Furthermore, recent studies suggest that OT and ET patients share some non‐motor clinical features (i.e., cognitive and neuropsychiatric changes, particularly those thought to rely on the integrity of the prefrontal cortex, which suggests involvement of frontocerebellar circuits).^3^, ^4^, ^14^, ^15^ In this sense, the pathogenesis of both OT and ET remains unclear, although clinical and neuroimaging data suggest that it may be related to the existence of alterations in the cerebello‐thalamo‐cortical network.^2^, ^7^, ^8^, ^16^, ^17^, ^18^

Irrespective of whether the two diseases are linked or not, OT is not widely recognized by physicians who are not movement disorders experts, which often results in misdiagnosis for the unfortunate patients, who then may be subjected to inappropriate or unnecessary tests and treatments.^9^, ^11^, ^19^ The differentiation of OT from ET may be therefore sometimes challenging.

In a recent volumetric MRI study involving 17 OT patients versus 17 controls,^20^ patients had bilateral decreased grey matter volume (atrophy) in the lateral cerebellum, and bilateral increased grey matter volume in the supplementary motor area and vermian grey matter. This would suggest the possibility of mild volumetric changes in OT. In this context, the width of the cortical grey matter layer, that covers the surface of the brain, referred to as cortical thickness that has been assessed by means of MRI, as useful measures in a variety of disorders to study neuroanatomical patterns, including ET,^21^, ^22^ but not in OT. The analysis of this biomarker, using statistical packages for neuroimaging analysis like SPM (http://www.fil.ion.ucl.ac.uk/spm/), FSL (https://fsl.fmrib.ox.ac.uk/fsl/fslwiki https://fsl.fmrib.ox.ac.uk/fsl/fslwiki https://fsl.fmrib.ox.ac.uk/fsl/fslwiki https://fsl.fmrib.ox.ac.uk/fsl/fslwiki), or FreeSurfer (http://surfer.nmr.mgh.harvard.edu), allows us to study differences between groups (e.g., OT vs. ET). However, these methods are not applicable on a single‐subject level and therefore do not improve the clinical diagnosis potential. To overcome this issue, data mining techniques have recently been identified as promising tools in neuroimaging data analysis for individual class prediction.^23^ Automatic classification techniques provide tools for analyzing all these variables simultaneously and observe inherent disease‐related patterns in the data.^23^

Even though data mining techniques have been widely used for MRI images in several neurological disorders,^21^, ^24^, ^25^, ^26^ no study to date has been conducted to differentiate between OT and ET patients. We hypothesized that a data mining approach, applied to MRI‐derived brain volume and cortical thickness data, could differentiate between OT and ET patients. The primary aim of this study was therefore to test and evaluate the effectiveness of data mining for single‐subject level classification of individuals affected by OT and ET. Toward this purpose, we used MRI‐derived brain volume and cortical thickness data from a cohort of OT and ET patients. As a secondary aim, in OT patients, we tested the effectiveness of data mining for single‐subject level classification of individuals affected by primary OT versus those with additional signs.

## Methods

All the participants included in the study gave their written informed consent after full explanation of the procedure. The study, which was conducted in accordance with the principles of the Helsinki declaration, was approved by the ethical standards committee on human experimentation at the University Hospital “12 de Octubre” (Madrid). Written (signed) informed consent was obtained from all enrollees.

### Participants

Patients with OT and ET were consecutively recruited from December 2011 to July 2013 from the outpatient neurology clinics of the University Hospital “12 de Octubre” in Madrid (Spain), a public hospital, which covers an area of more than 400,000 inhabitants. Three neurologists with expertise in movement disorders (J.B.‐L., A.S.‐F., and M.M.) examined OT patients. In OT patients, the neurological examination comprised a general neurological examination and the motor portion of the Unified Parkinson’s Disease Rating Scale (m‐UPDRS).^27^ Mild parkinsonian signs were defined as present when any one of the following conditions was met: (1) two or more m‐UPDRS^27^ ratings = 1; (2) one m‐UPDRS^27^ rating = 2; or (3) the m‐UPDRS rest tremor rating = 1.^28^ Diagnoses of OT were assigned by the four neurologists using the Consensus Statement on Tremor by the Movement Disorder Society.^29^

OT patients were 1:1 frequency matched with ET patients. Frequency matching was based on right handedness, age, sex, and years of education.

In the case of ET patients, a 20‐min, semi‐structured, tremor interview was conducted in which demographic information and data on tremor (e.g., duration) were collected. Two neurologists with expertise in movement disorders (J.B.‐L. and J.P.R.) examined the patients and used the Fahn‐Tolosa‐Marìn tremor rating scale to assign a total tremor score (range = 0–144).^30^ Diagnoses of ET were assigned by the two neurologists using the Consensus Statement on Tremor by the Movement Disorder Society.^29^

### Procedure

Clinical characteristics were obtained from review of records from their outpatient neurological care. All the neuropsychological tests were performed on the same day by the same examiner (V.P., see acknowledgments). All participants underwent a neuropsychological assessment of cognitive functioning (Table 1).^15^, ^31^, ^32^ The tests chosen for the battery attempted to make minimal demands on motor processes to avoid the effects of any hand tremor.^15^ Severity of depressive symptoms was measured by the 17‐item version of the Hamilton Depression Rating Scale.^33^

**Table 1:** Comparison of demographic, clinical, and neuropsychiatric domains of orthostatic tremor patients vs. essential tremor patients.

	Orthostatic tremor patients (*N* = 14)	Essential tremor patients (*N* = 15)	*P* value
Age in years	65.0 (66.9) ± 13.9	68.5 (69.0) ± 8.3	0.417^a^
Sex (female)	12 (52.2%)	11 (47.8%)	0.411
Education in years	8.0 (8.0) ± 4.9	6.9 (8.0) ± 3.1	0.621^b^
Tremor duration, years	9.4 (7.0) ± 6.9	24.9 (18.0) ± 18.4	0.003^b^
Cognitive domains			
Attention			
Direct digit span test from the WAIS‐III	5.2 (5.0) ± 1.5	5.1 (5.0) ± 1.2	0.880^b^
WAIS‐III digit symbol – coding subtest	39.0 (28.5) ± 31.4	29.7 (24.0) ± 15.4	0.943^b^
Executive functions			
Stroop color – word trial	24.6 (22.0) ± 13.9	25.5 (27.0) ± 10.8	0.854^a^
Frontal assessment battery	14.3 (15.0) ± 3.2	13.7 (15.0) ± 5.1	0.964^b^
Indirect digit span test from the WAIS‐III	3.1 (3.0) ± 1.3	3.5 (3.0) ± 1.1	0.591^b^
Controlled oral word association test	26.1 (26.5) ± 21.8	24.7 (20.0) ± 13.9	0.828^a^
Visual memory			
Brief visuospatial memory test‐revised			
Learning total	15.1 (10.5) ± 12.3	21.4 (19.0) ± 9.8	0.136^a^
Delayed free recall trial	5.5 (4.5) ± 4.8	8.4 (10.0) ± 3.6	0.123^b^
Recognition trial	11.7 (12.0) ± 0.5	11.7 (12.0) ± 0.6	0.949^b^
Visuospatial ability			
Benton judgment of line orientation test	8.3 (8.5) ± 3.2	9.0 (9.0) ± 2.7	0.560^a^
Hooper visual organization test	28.6 (29.0) ± 14.3	34.0 (35.0) ± 9.6	0.247^a^
Verbal memory			
WMS‐III word list			
Learning list	26.3 (24.0) ± 7.2	27.9 (27.0) ± 5.1	0.481^a^
Immediate recall	5.0 (4.5) ± 2.4	6.0 (6.0) ± 2.0	0.234^a^
Delayed recall	4.6 (4.0) ± 2.8	5.5 ± (6.0) 2.3	0.397^a^
Recognition	19.8 (20.5) ± 4.0	22.3 (22.0) ± 1.5	0.077^b^
Language			
Boston naming test	40.2 (36.0) ± 11.3	41.1 (44.0) ± 12.7	0.863^a^
Depressive symptoms			
17‐item Hamilton Depression Rating Scale total score	7.1 (6.0) ± 6.7	8.2 (8.0) ± 5.1	0.615^a^

Mean (median) ± standard deviation and frequency (%) are reported. WAIS‐III, Wechsler Adult Intelligence Scale‐Third Edition; WMS‐III, Wechsler Memory Scale‐Third Edition.

a Student’s *t*‐test.

b Mann–Whitney test were used for comparisons of continuous data, and *X* ^2^ test for proportions.

### Neuroimage acquisition

Participants were immobilized with a custom‐fit blue bag vacuum mold (Medical Intelligence, Inc.) to prevent image artifacts. A strict criterion for head movement assessment was adopted (maximal absolute head movement less than 1.0 mm and 1.0° in the *x*‐, *y*‐, and *z*‐directions) and neither OT patients nor ET patients were excluded from the analysis due to this criterion. All MRI data were acquired with a clinical 3T Signa HDx MRI scanner (GE Healthcare, Waukesha, WI) using an 8‐channel phased array coil. The imaging (MRI) standardized protocol included a 3D T1‐weighted SPGR with a TR  = 9.2 msec, TE  = 4.128 msec, TI = 500 msec, NEX = 1, acquisition matrix = 240 × 240, full brain coverage, resolution = 0.9375 × 0.9375  × 1 mm, and flip angle = 12.

### Neuroimage processing

MRI images were processed to extract volumetric and cortical thickness features, which were calculated using the freely available software FreeSurfer (http://surfer.nmr.mgh.harvard.edu/). Using a surface‐based approach, FreeSurfer can automatically segment the brain into different cortical and subcortical regions of interest and calculate average thickness in the defined regions. Briefly, FreeSurfer's main cortical reconstruction pipeline began with the registration of the structural volume with the Talairach atlas.^34^ After bias field estimations and the removal of this bias, the skull was stripped of non‐brain tissue using a hybrid watershed/surface deformation procedure and subcortical white and grey matter structures were segmented.^35^, ^36^ Next, tessellation of the gray matter and white matter boundary, automated topology correction, and surface deformation routines were used to create the white/grey (white) and grey/cerebrospinal fluid (pial) surface models.^37^ These surface models were then inflated, registered to a spherical atlas, and used to parcellate the cortical mantle, according to gyral and sulci curvature.^38^ This method used both intensity and continuity information from the entire three‐dimensional MRI volume in segmentation and deformation procedures to produce representations of cortical thickness, calculated as the closest distance from the gray/white boundary to the gray/CSF (cerebrospinal fluid) boundary at each vertex on the tessellated surface.^39^ The cortical thickness features were average values for each region. Additionally, for each cortical region, the standard deviation of the cortical thickness was also calculated as a measure of roughness. We should keep in mind that the distribution of cortex thickness is not uniform by layer, neither is the variation in the thickness of the cortical layers proportional to the variation in the total thickness nor is the location and progression of subtle cortical atrophy the same among individuals with the same neurodegenerative disease.^21^, ^22^, ^40^ Hence, there is also a need for new and more reliable variables to analyze the pattern of cortical thickness.^21^ “Roughness” within a certain area may therefore be a promising metric to overcome these limitations.^21^, ^22^, ^40^ An increase in roughness is usually associated with cortical thinning (i.e., atrophy).^21^, ^22^, ^40^

A total of 281 attributes from each participant resulted from the above processing analysis. Volumetric features were normalized using both the total intracranial volume and the total grey matter volume. The accuracy of FreeSurfer's results was then assessed visually for the different participants.

### Statistical analyses of clinical and neuropsychological data

Statistical analyses for the clinical and neuropsychological measures were conducted using Statistical Package for the Social Sciences (SPSS) Version 25.0 (SPSS, IBM Corporation). Demographic and clinical variables were compared using two independent sample *t*‐tests for continuous and normally distributed data, and Mann–Whitney *U* test for non‐normally distributed data, where appropriate. The *χ* ^2^ test was used to analyze differences in sex distribution.

The differences in MRI variables between the two groups were analyzed using an ANCOVA test, taking as covariates age, sex, education, and tremor duration.

To assess differences between OT and ET patients in neuropsychological scores while adjusting for age, sex, years of education, and depressive symptoms, linear regression analyses were performed in which the outcome variables were each one of the neuropsychological scores.

Differences were considered statistically significant for *P* values <0.05.

### Feature selection and classification

The MRI variables that presented significant differences between the two groups were scored by the information gain ratio measure (IGR).^41^ IGR was chosen as most appropriate type of feature weighting (Information Theory‐based), given the study sample structure and size.^42^ More specifically, there were a small number of participants who were then further divided into additional categories (14 OT vs. 15 ET). Furthermore, each participant was characterized by a high number of independent variables (281 attributes from the processing analysis).

The variables were then sorted by this measure in descending order. After that, a 10 cross‐fold validation with a Support Vector Machine (SVM) classifier (dot kernel, kernel cache = 2000, complexity constant = 0.0, convergence epsilon = 0.001) was iteratively applied to a subset of the MRI variables scored.^43^ The subset was first formed by the variable with the highest IGR. Then, the classifier validation was performed for that subset. Later, the variable with the second highest IGR was added to the subset. If the classification accuracy from the 10 cross‐fold validation was equal or higher than the one obtained with the previous subset, the added variable was kept. Otherwise, the variable was discarded and the variable with the next highest IGR was added to the subset. The process stopped when all the variables were added to the subset and tested. At the end of the process, a maximum subset of MRI variables producing the best classification results was obtained.

We did not choose other common feature selection approaches, such as factor analysis (principal component analysis and independent component analysis), regression analysis (linear or binary logistic), or complete rank selection due to the small size of the sample and the high number of independent variables. These other approaches of selection would likely have yielded meaningless models overfitting the data and, consequently, with no prediction capacity.

## Results

Clinical details of the OT patients have been published elsewhere.^31^, ^32^ Briefly, all 14 OT patients were right‐handed (mean age 65.0 years, range 37–81 years). There was a female preponderance (N = 12, 85.7%) with a mean age of onset of 55.6 years. Nine (64.3%) patients presented with primary OT and five patients (35.7%) had additional neurological features (mild parkinsonian signs). Structural brain MRI was unremarkable in all 14 OT patients, none had cerebellar atrophy.

The 14 right‐handed OT patients were compared with 15 right‐handed ET patients (11 women and 4 men). The mean total tremor score of ET patients was 35.7 ± 14.6 (median 34.0). The OT patients did not differ to a significant degree from the ET patients in terms of age, sex, years of education, and depressive symptoms (Table 1). However, the tremor duration was significantly higher in OT patients than in ET patients. The results of neuropsychological testing are shown in Table 1. In all domains, OT patients’ cognitive performance did not differ from ET patients (Table 1).

Although OT patients and ET patients did not differ to a significant degree in terms of demographic features, if the sample size had been larger, several of these features could have differed significantly. Hence, we performed adjusted analyses to take any potential confounding into account. In linear regression analyses that adjusted for age, sex, years of education, depressive symptoms, and tremor duration, we found that diagnosis (OT vs. ET) was not associated with poor performance on any neuropsychological test scores.

### Comparison of MRI‐derived brain volume and cortical thickness areas between OT and ET patients

Given that the tremor duration showed a statistically significant difference between the two groups (OT vs. ET), the analyses of differences of MRI variables were corrected by tremor duration, along with age, sex, and years of education. Table 2 presents only the statistically significant differences between the two groups after correcting by those variables. Bilateral thalamus and basal ganglia volume appeared reduced in the ET group compared with the OT group. Bilateral parietal areas in the ET group were less uniform, in terms of cortical thickness, than in the OT group. Finally, right parietal and posterior cingulate cortical areas were thicker in the ET group than in the OT group.

**Table 2:** Statistically significant differences in MRI‐derived brain volume and cortical thickness between groups, adjusted for age, sex, education, and tremor duration.

Structural MRI variable	ET‐OT difference	Statistic
L thalamus proper volume/GM	−0.00201	F(1,23) = 13.084, *P* = 0.001, *η* ^2^ = 0.363
L thalamus proper volume/ICV	−0.00099	F(1,23) = 22.923, *P* < 0.0001, *η* ^2^ = 0.499
L putamen volume/ICV	−0.00054	F(1,23) = 7.589, *P* = 0.011, *η* ^2^ = 0.248
L hippocampus volume/GM	−0.00084	F(1,23) = 5.190, *P* = 0.032, *η* ^2^ = 0.184
L hippocampus volume/ICV	−0.00047	F(1,23) = 12.168, *P* = 0.002, *η* ^2^ = 0.346
R thalamus proper volume/ICV	−0.00060	F(1,23) = 8.258, *P* = 0.009, *η* ^2^ = 0.264
R caudate volume/ICV	−0.00038	F(1,23) = 6.519, *P* = 0.018, *η* ^2^ = 0.221
R putamen volume/GM	−0.00109	F(1,23) = 14.384, *P* = 0.001, *η* ^2^ = 0.385
R putamen volume/ICV	−0.00060	F(1,23) = 19.781, *P* < 0.0001, *η* ^2^ = 0.462
R pallidum volume/ICV	−0.00015	F(1,23) = 7.841, *P* = 0.010, *η* ^2^ = 0.254
R hippocampus volume/ICV	−0.00039	F(1,23) = 8.342, *P* = 0.008, *η* ^2^ = 0.266
ICV	184623.56089	F(1,23) = 11.589, *P* = 0.002, *η* ^2^ = 0.335
L fusiform volumen	−802.67619	F(1,23) = 7.342, *P* = 0.013, *η* ^2^ = 0.242
L pars opercularis thickness	0.10483	F(1,23) = 5.801, *P* = 0.024, *η* ^2^ = 0.201
L lingual thickness	0.12534	F(1,23) = 6.347, *P* = 0.019, *η* ^2^ = 0.216
L pars opercularis roughness	0.06729	F(1,23) = 4.839, *P* = 0.038, *η* ^2^ = 0.174
L superior parietal roughness	0.10372	F(1,23) = 8.456, *P* = 0.008, *η* ^2^ = 0.269
L inferior parietal roughness	0.11265	F(1,23) = 8.287, *P* = 0.008, *η* ^2^ = 0.265
L precuneus roughness	0.09910	F(1,23) = 9.248, *P* = 0.006, *η* ^2^ = 0.287
R superior parietal volume	5867.17619	F(1,23) = 62.627, *P* < 0.0001, *η* ^2^ = 0.731
R superior parietal thickness	0.46694	F(1,23) = 41.654, *P* < 0.0001, *η* ^2^ = 0.644
R middle temporal thickness	0.20840	F(1,23) = 4.506, *P* = 0.045, *η* ^2^ = 0.164
R isthmus cingulate thickness	0.17141	F(1,23) = 5.698, *P* = 0.026, *η* ^2^ = 0.199
R superior parietal roughness	0.09128	F(1,23) = 9.634, *P* = 0.005, *η* ^2^ = 0.295
R inferior parietal roughness	0.10284	F(1,23) = 26.297, *P* < 0.0001, *η* ^2^ = 0.533

GM, Total Grey Matter volume; ICV, Total Intracranial Volume; L, Left hemisphere; R, Right hemisphere; *η* ^2^, Effect size.

### Primary Aim: Feature selection and classification (OT vs. ET)

**Figure 1:**
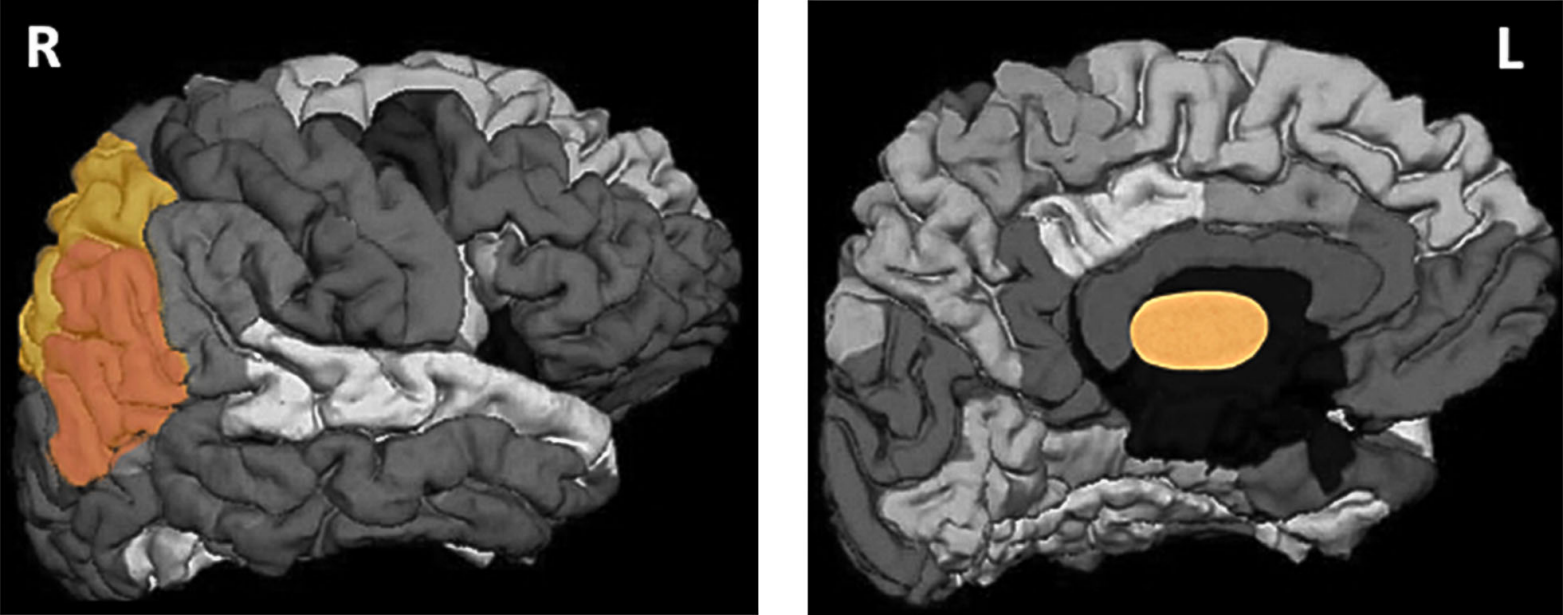
Regions of interest in the Desikan‐Killiany atlas yielding the best classification accuracy between orthostatic and essential tremor groups. R: Right hemisphere; L: Left hemisphere.

**Figure 2:**
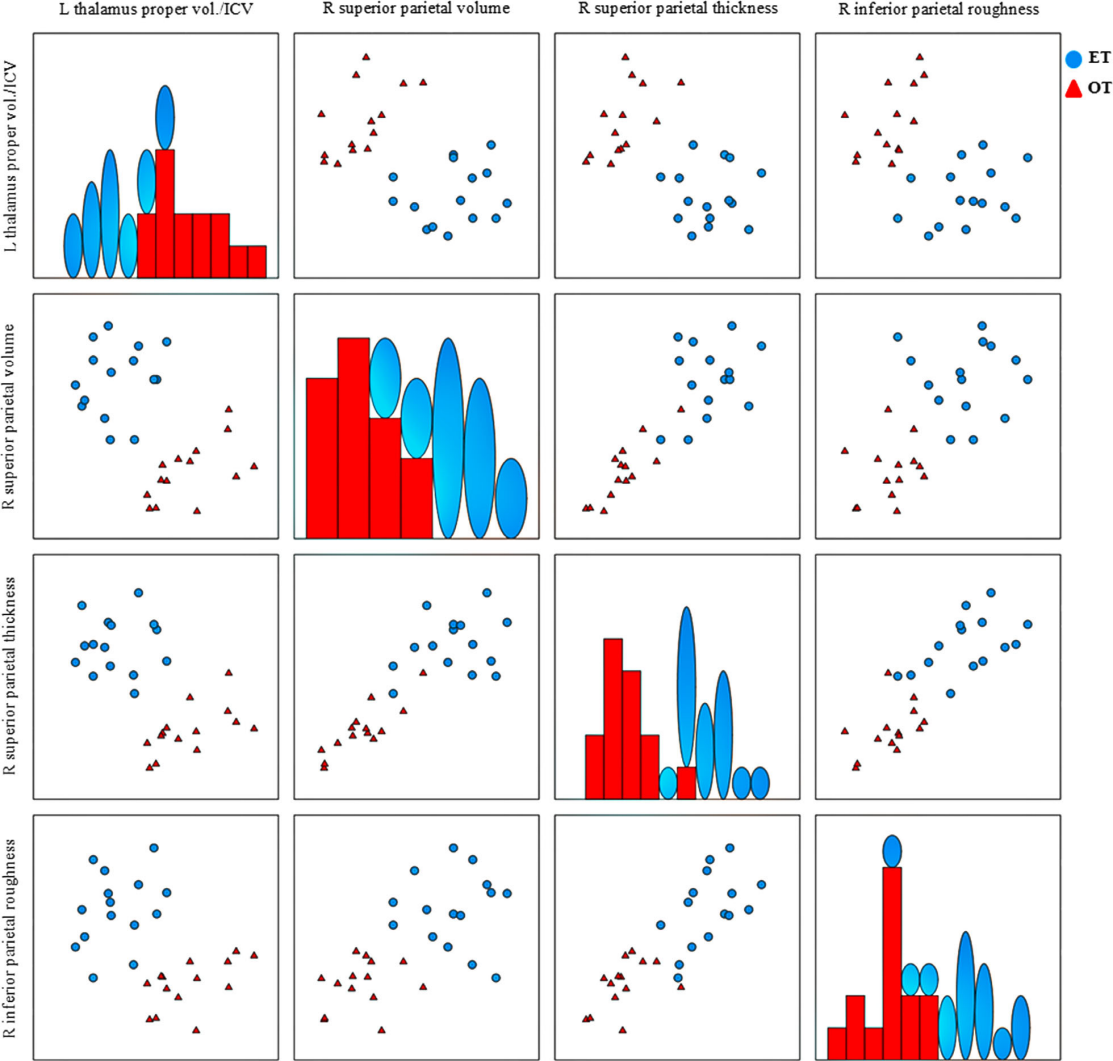
Scatter matrix of the variables forming the best classification subset to distinguish between orthostatic and essential tremor groups.

After the feature selection process, a subset of four MRI variables formed by the left thalamus proper volume (normalized by the total intracranial volume), right superior parietal volume, right superior parietal thickness, and right inferior parietal roughness yielded a classification accuracy of 100%. Figure 1 shows the regions of interest corresponding to the variables in the subset according to the Desikan‐Killiany atlas. Figure 2 shows the scatter matrix of the variables forming the best classification subset for the two groups. Graphs in the diagonal show accumulated histograms of the corresponding variables for the two groups. In general terms, the ET group presented a lower left thalamus proper volume and higher right superior parietal volume and thickness and increased inferior parietal roughness.

**Figure 3:**
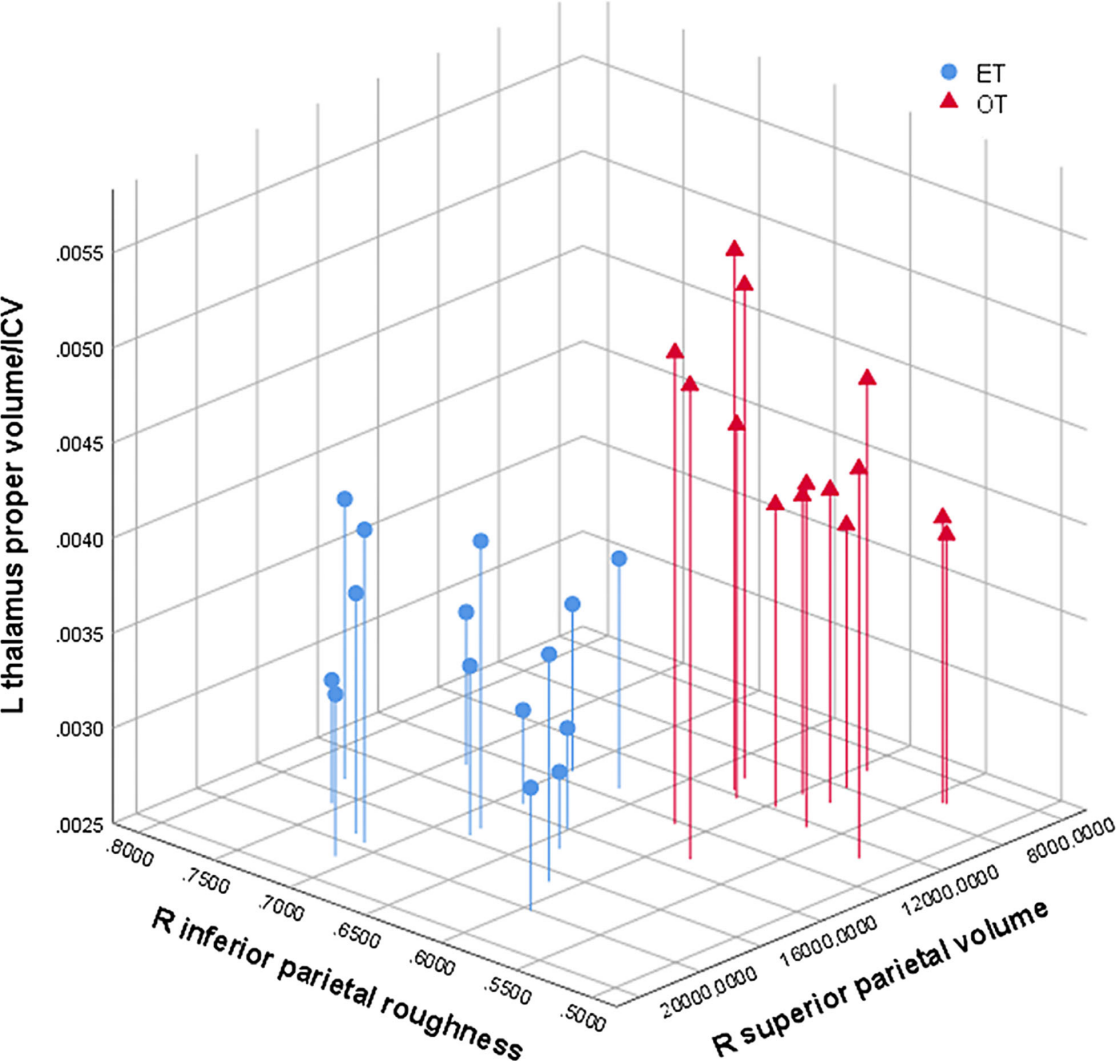
Scatter plot of the three MRI variables that produce the highest linear separation between the orthostatic and essential tremor groups.

The combination of the left thalamus proper volume (normalized by the total intracranial volume) with any of the other cortical parietal variables produced a linear separation between the two groups (Fig. 2, row 1). Additionally, the combination of right superior parietal volume and right inferior parietal roughness also resulted in a linear separation of the two groups. The combination of these three variables was the one that produced the highest linear separation, in Euclidean distance terms, between the two groups, as shown in Figure 3.

### Comparison of MRI‐derived brain volume and cortical thickness areas between primary OT and OT patients with mild parkinsonian signs

The primary OT patients did not differ from those OT patients with mild parkinsonian signs in terms of sex, years of education, and tremor duration (Table 3). However, they were younger (Table 3). The statistically significant differences in MRI‐derived brain volume and cortical thickness variables between primary OT and OT patients with mild parkinsonian signs are shown in Table 3 after having corrected by age, sex, years of education, and tremor duration. Essentially, the right pallidum showed a higher volume in primary OT than in those with mild parkinsonian signs. However, primary OT showed a thinner left caudal anterior cingulate and higher left caudal middle frontal roughness than OT patients with mild parkinsonian signs. Despite the corrections, the right pallidum volume presented a strong correlation with age (Pearson’s rho = −0.736, *P* = 0.003).

**Table 3:** Comparison of demographic, clinical, and MRI features of primary orthostatic tremor patients vs. those with mild parkinsonian signs.

	Primary orthostatic tremor patients (*N* = 9)	Orthostatic tremor patients with mild parkinsonian signs (*N* = 5)	*P* value
Age in years	59.0 (61.9) ± 13.8	75.7 (76.5) ± 4.6	0.024^a^
Sex (female)	7 (77.8%)	5 (100.0%)	0.255^a^
Education in years	9.8 (9.0) ± 4.9	4.8 (4.0) ± 3.3	0.07^a^
Tremor duration, years	9.2 (6.4) ± 8.4	9.7 (8.5) ± 3.6	0.900^a^

Structural MRI variable	Primary OT‐OT with mild parkinsonian signs difference	Statistic
R pallidum volumen	360.54222	F(1,8) = 8.398, *P* = 0.020, *η* ^2^ = 0.512
R pallidum volumen/GM	0.00045	F(1,8) = 14.217, *P* = 0.005, *η* ^2^ = 0.640
R pallidum volumen/ICV	0.00017	F(1,8) = 7.349, *P* = 0.027, *η* ^2^ = 0.479
L caudal anterior cingulate thickness	−0.49968	F(1,8) = 12.939, *P* = 0.007, *η* ^2^ = 0.618
L caudal middle frontal roughness	0.03900	F(1,8) = 5.359, *P* = 0.049, *η* ^2^ = 0.401

Mean (median) ± standard deviation and frequency (%) are reported. GM, Total Grey Matter volume; ICV, Total Intracranial Volume; L, Left hemisphere; R, Right hemisphere; η^2^: Effect size.

a Student’s *t*‐test for continuous data, and *X* ^2^ test for sex.

### Secondary Aim: Feature selection and classification (primary OT vs. OT with mild parkinsonian signs)

**Figure 4:**
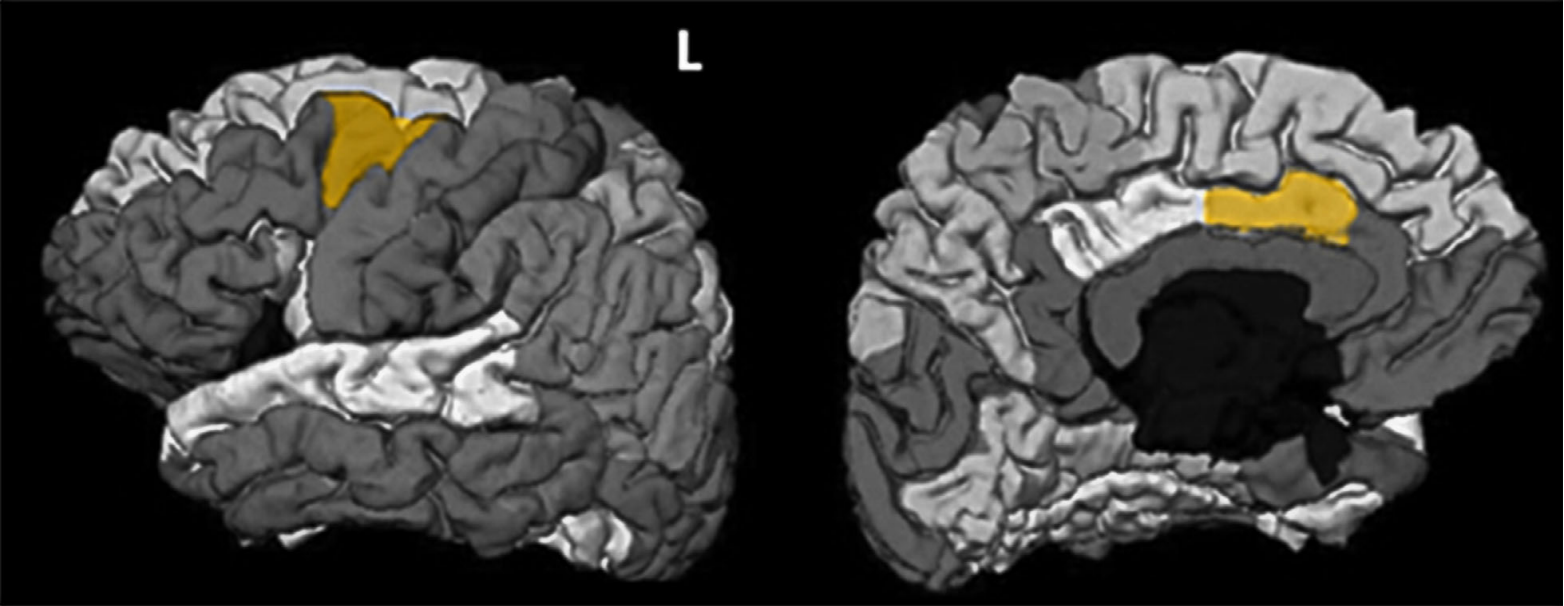
Regions of interest in the Desikan‐Killiany atlas yielding the best classification accuracy between primary orthostatic and those with mild parkinsonian signs essential. L: Left hemisphere.

From the MRI variables in Table 3, a subset composed of two variables provided a classification accuracy of 100% – the left caudal anterior cingulate thickness and the left caudal middle frontal roughness (see Fig. 4).

**Figure 5:**
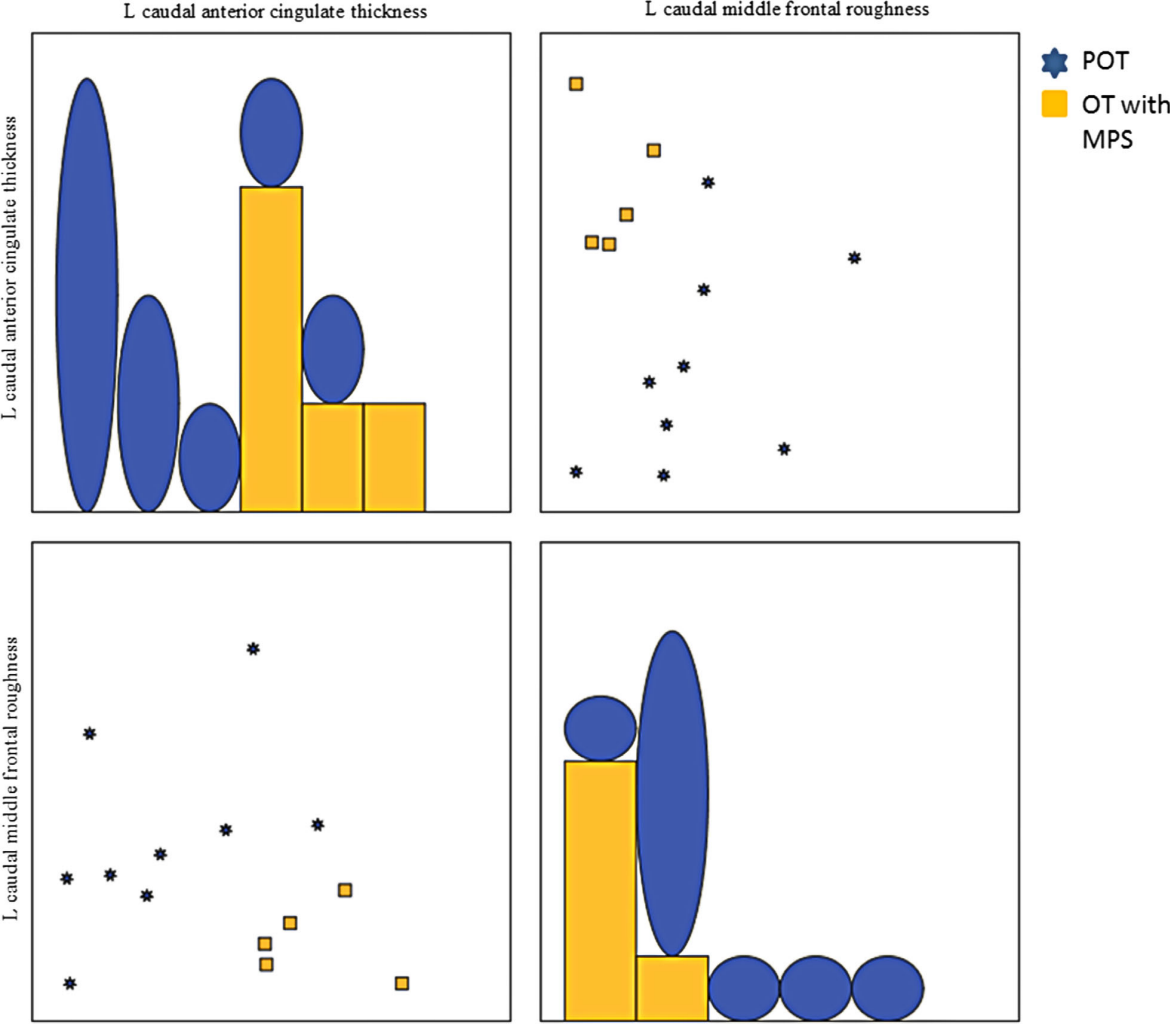
Scatter matrix of the two MRI variables that produce the highest linear separation between primary orthostatic and those with mild parkinsonian signs.

Analogously to ET‐OT classification, Figure 5 shows the scatter matrix of the variables forming the best classification subset for the two OT groups. Graphs in the diagonal show accumulated histograms of the corresponding variables for the two groups. In general terms, the primary OT group presented a lower left caudal anterior cingulate thickness and an increased left caudal middle frontal roughness, pointing to a deterioration of those areas. Both variables allowed us a linear separation between the two groups.

## Discussion

Efforts to improve the characterization of OT are not only essential to prevent underdiagnosis but also to enhance medical counselling, surveillance recommendations, and future treatment strategies. Apart from the debate that OT may be a variant of ET or that they may coexist, both disorders are occasionally misdiagnosed between them and they share some non‐motor symptoms (e.g., depression, personality changes, and cognitive dysfunction).^3^, ^4^, ^14^, ^15^ In this sense, we did not find there were any differences between them in the studied cognitive areas (see Table 1).

The data mining techniques applied, forward wrapper selection from information gain ranking filter and exhaustive subset wrapper search, have been applied individually to biomedical data, but never in combination.^44^ The novelty of the current study is that we used them together and applied them to MRI data for the first time. In fact, no previous studies have tried to differentiate OT from ET with data mining techniques using MRI‐derived brain volume and cortical thickness. This approach not only allowed us to differentiate both disorders but also to understand relative contributions of factors for distinguishing these tremor‐defined syndromes, which suggests that they are not variant or related disorders. These results also shed light on a more dimensional understanding of the neurobiological variables that contribute to these disorders.

Our analysis of MRI showed that four features were relevant for the classification process (OT vs. ET): the left thalamus proper volume (normalized by the total intracranial volume), right superior parietal volume, right superior parietal thickness, and right inferior parietal roughness. Although the parietal cortex may be also affected in ET,^21^, ^22^ we had previously reported a significant decrease in the absolute concentration of N‐acetylaspartate + N‐acetylaspartyl glutamate in OT patients in midparietal gray matter, suggesting that there is neuronal damage or loss in OT, raising the intriguing question as to whether it is a neurodegenerative disease.^32^ On the other hand, two features were relevant for the classification process (primary OT vs. OT with mild parkinsonian signs): the left caudal anterior cingulate thickness and the left caudal middle frontal roughness. Overall, this suggests that the pathogenesis of primary OT may differ from that of OT with associated features. Moreover, the deterioration in primary OT of the caudal anterior cingulate area is shared with other conditions that involve dopaminergic deficits, such as schizophrenia,^45^ obsessive‐compulsive disorder,^46^ and Parkinson’s disease.^47^ This common factor would point to the hypothesis of a dopaminergic role in the pathogenesis of primary OT, rather than the central oscillatory network or altered cerebello‐thalamo‐cortical network.^48^ More research is however needed to establish these statements.

The comparison with other studies in related disorders is not easy, since they describe a wide range of different accuracies for classification and prediction tasks and have used different features and techniques. Feature extraction methods, feature selection or classification tools, neuroimage quality, number of participants, and demographics are also crucial considerations. Our classification accuracies – 100% in distinguishing OT from ET patients, and 100% in differentiating subgroups of OT agree with previous ones based on data mining techniques in other tremor disorders designed to differentiate these ones form healthy controls, such as Parkinson’s disease, where the accuracy tends to be approximately 80%.^26^ They are also similar to others designed to discriminate Parkinson’s disease from progressive supranuclear palsy (accuracy > 90%),^49^ or to separate Parkinson’s disease patients with and without mild cognitive impairment (accuracy of 80%).^50^ Notwithstanding, there remains much to learn about these techniques in other tremor diseases, especially in OT.

The study should be interpreted within the context of several limitations. The most important is the small sample size. Given the low prevalence and incidence of the disease, the OT literature generally comprises studies with small sample sizes.^2^, ^48^ However, we could classify with an overall accuracy of 100% between OT and ET patients even with these small numbers. Notwithstanding, these findings should be taken cautiously until they are replicated in larger and independent data sets (multi‐center and/or multiscanner).

In summary, we have shown that a data mining approach applied to MRI‐derived brain volume and cortical thickness data may differentiate between two tremor disorders (OT and ET), with accuracy of 100%. From a clinical point of view, it is extremely important to differentiate both entities (OT vs. ET), since treatment approach is different. Although this is the first application of data mining techniques to the classification of OT patients, the scores obtained are in accordance with previous tremor studies. This technique has the potential to be used to research in tremor diseases.

## Authors Contributions

Benito‐León (jbenitol67@gmail.com) collaborated in (1) the conception, organization, and execution of the research project; (2) the statistical analysis design, and (3) the writing of the manuscript first draft and the review and critique of the manuscript. Louis (elan.louis@yale.edu) collaborated in (1) the conception and organization of the research project and (2) the review and critique of the manuscript. Mato‐Abad (virginia.mato@urjc.es) collaborated in (1) the conception and organization of the research project and (2) the review and critique of the manuscript. Sánchez‐Ferro (alvarosferro@hotmail.com) collaborated in (1) the organization of the research project and (2) the review and critique of the manuscript. Romero (p.romero.prof@ufv.es) collaborated in (1) the conception and organization of the research project and (2) the review and critique of the manuscript. Matarazzo (michele.matarazzo@gmail.com) collaborated in (1) the organization of the research project and (2) the review and critique of the manuscript. Serrano (jignacio.serrano@csic.es) collaborated in (1) the conception, organization, and execution of the research project; (2) the statistical analysis and design, and (3) the review and critique of the manuscript.

## Conflict of Interest

The authors declare no competing financial interests.
